# On the structure of psychoeducational constructs: taxometric analysis and epistemological implications

**DOI:** 10.3389/fpsyg.2025.1499960

**Published:** 2025-03-17

**Authors:** Dimitrios Stamovlasis, Julie Vaiopoulou, Georgia Stavropoulou, Theano Papagiannopoulou

**Affiliations:** ^1^Department of Philosophy and Education, Aristotle University of Thessaloniki, Thessaloniki, Greece; ^2^School of Humanities, Hellenic Open University, Patras, Greece; ^3^Department of Education, University of Nicosia, Nicosia, Cyprus

**Keywords:** latent constructs, taxometrics, taxon, dimensional, MAMBAC, MAXEIG, L-Mode, comparison curve fit index

## Abstract

Taxometric analysis (TA) is a technique designed to elucidate the structure of a psychological construct, specifically determining whether the latent variable is categorical (taxon) or dimensional. The taxon hypothesis is significant because the structure of a latent construct influences how we conceptualize, characterize, and measure it, thereby impacting the methodologies employed in both research and practical applications. In this study, data from two separate studies were subjected to TA. Study 1 involves secondary school students (*N* = 2024) and explores factors such as Achievement Goals and Self-Efficacy within the context of language acquisition. Study 2 examines issues among service teachers (*N* = 494) and includes variables such as Attitudes, Self-Efficacy, Commitment, and Cognitive and Affective conditions within the framework of STEM education. Given that the taxon hypothesis is tested for the first time using these types of psychoeducational data, Taxometrics is applied in an exploratory manner to provide a deeper understanding of the nature of these constructs. The results of TA are based on a series of indicators that identified cases of dimensional constructs when items from a single dimension were used as input. However, when all elements related to achievement goals and teacher readiness were utilized as input, the results revealed ambiguous latent structures. This emerging ambiguity prompts theoretical and epistemological discourse to explain the findings and advocate for a reevaluation of the nature of latent psychoeducational constructs.

## 1 Introduction

Research in psychology and education presupposes the measurement of individual differences, attributes, traits, and, in general, the factors involved in these studies. Access to these variables poses challenges because the ontology of these intangible entities remains undefined and vague. However, by using mathematical models, it becomes possible to establish probabilistic relationships between latent theoretical constructs and their empirical indicators, assuming that these hypothetical latent entities are common causes of their observable manifestations. The researcher can then implement a psychometric model based on theoretical assumptions, such as a factor model, IRT, latent profile, or latent class model (Bartholomew, 1987). The recommended modeling approach involves utilizing various psychometric models and selecting the most suitable one based on a comparison of fit indices (Lubke and Neale, 2006, 2008; Lubke and Miller, 2015). In certain cases, the choice of psychometric model is guided by the theoretical foundations of specific phenomena. For instance, when studying mental models in science learning, the most suitable model to employ is the latent class model (Stamovlasis et al., 2018).

The nature of the latent construct can also be posed as a research question, which can be investigated through *taxometric analysis* (TA). While known psychometric approaches estimate structural model parameters, Taxometrics serves as the method to detect the type of structural model (McGrath and Walters, 2012). The idea of TA originally emerged from psychiatric research and was specifically designed to identify and categorize distinct latent classes of behavioral traits or syndromes. The question of whether latent variables should be treated as discrete entities or continua is of paramount importance (Frazier et al., 2010; Edens et al., 2011; Haslam et al., 2012). The implications concern their definition and measurement-diagnosis procedures, and it has, of course, significant consequences for treatment, while early identification can also improve understanding of their etiology. Moreover, TA is a crucial entreaty that pertains to theory and methodology development. Taxometric procedures are based on the observation of the effects of the posited model on the statistical relations of observable indicators (Waller and Meehl, 1998; De Boeck et al., 2005; Schmitt et al., 2006; Lubke and Miller, 2015).

Taxometrics has been a popular and useful method in medicine and psychopathology and has contributed a lot in revealing the nature of many latent causes associated with various symptoms (Meehl, 1995; Haslam et al., 2012, 2020). TA appeared more useful in psychiatric research, specifically where tool development, classification, and diagnosis (e.g., DSM-V; American Psychiatric Association, 2013; Widiger and Samuel, 2005) acquired an additional advantage with taxon characterization, which has been applied in searching various psychopathologies, such as autistic disorders addictions, and schizotypy (Cuesta et al., 2007; Rawlings et al., 2008; James et al., 2016). TA has also been applied to personality research, clinical psychology, and antisocial behavior (Walters, 2011). In other fields of behavioral sciences, TA is less commonly used, and this sets off new exploratory routes of investigation.

### 1.1 The present research: rationale and the research hypothesis

The mainstream of psychoeducational research has not used Taxometrics compared to the psychiatric domain, which also addresses latent constructs and shares similar methodologies. In psychoeducational research, except for cases such as exploring mental models, where LCA is intentionally employed due to theoretical presuppositions (Stamovlasis et al., 2018), both factor models and latent class analysis are typically used alternatively, with the best-fit model being retained. However, it is important to note that many continuous-variable models have statistically equivalent categorical or mixture alternatives (Halpin et al., 2011). Thus, the nature of the variable in question remains unknown, which is not merely a theoretical concern. The taxon hypothesis will enhance understanding of the function of latent constructs and is essential for cognitive, attitudinal, and developmental variables, as the nature of these variables relates to pathways of change. Psychology and education are interested in such changes, including conceptual change, shifts in attitudes, and specific or general developmental outcomes. The mode or pathways of change are associated with the structure of the latent variable, which means that if the variable is dimensional, changes occur in a smooth linear manner, whereas if it is a taxon, changes are expected to be non-linear and discontinuous, occurring as transitions between levels or behavioral modes (e.g., van der Maas and Molenaar, 1992).

However, exploring the taxon hypothesis in psychoeducational research involves not only measurement but also the underlying theory, and it may have epistemological implications that could open new directions in psychometrics. This endeavor applies *TA* to data drawn from previously published work, which includes psychoeducational constructs such as achievement goals (mastery approach, performance approach, and performance avoidance), self-efficacy, commitment, and cognitive and affective conditions. The findings are expected to enhance understanding of the variables under investigation and potentially initiate a discussion on both measurement and theoretical issues.

The usefulness of identifying the nature of latent variables, as described in previous sections, provides a strong rationale for applying Taxometrics. Since there are very few contributions in psychoeducational research concerning the *taxon hypothesis*, it remains a fascinating and novel endeavor to investigate selected common latent variables related to students’ achievement goal orientations, self-efficacy, and teachers’ readiness for STEM education. These constructs were chosen because they are among the most well-researched in the literature and the foremost determinants of educational processes; their structure influences how changes occur and are observed. If it is continuous, changes occur linearly along the scale, whereas if it is a taxon, changes are expected to happen as discontinuous shifts between categories. The anticipated findings will enhance our understanding of the nature of the variables under investigation and possibly introduce new elements that could enrich psychometrics. Moreover, discussions on measurement, epistemological, and theoretical issues could be initiated.

## 2 Materials and methods

The data originated from two empirical studies. Study 1 examined students’ achievement goals, interests, and self-efficacy in language learning (Stavropoulou et al., 2023). Study 2 explored teachers’ readiness to implement STEM education (Papagiannopoulou et al., 2023). In both studies, the guidelines set by the Ethics and Deontology Committee were adhered to.

### 2.1 Data and procedures

#### 2.1.1 Study 1

##### 2.1.1.1 Sample

This study’s participants were high school students (*N* = 2045) aged 13 to 17 years. Of the participants, 50.2% were boys, and 49.8% were girls. They attended schools in Northern Greece and were of different socioeconomic statuses and living conditions.

##### 2.1.1.2 Measurements

Data collection was conducted using a paper-and-pencil procedure with the Patterns of Adaptive Learning Surveys scale (Midgley et al., 1998). The dimensions with their reliability measures were as follows: Master Approach (*α = 0.*83/*ω* = 0.82), Performance Approach (*α* = 0.82/*ω* = 0.82), and Performance Avoidance (*α* = 0.58/*ω* = 0.58). The fit indices for the three-factor model were satisfactory [*χ^2^_(101)_ =* 577.31; *p < 0*.001, *TLI* = 0.960; *CFI* = 0.996; *RMSEA* = 0.050 with *90% CI* = [0.046; 0.054]; *SRMR* = 0.049; *GFI* = 0.988; *NFI* = 0.960]. Self-Efficacy [Cronbach (*α* = 0.90/*ω* = 0.90); 9-item unidimensional scale with fit indices: *χ^2^*_(27)_ = 81.067, *p* < 0.001, TLI = 0.995, CFI = 0.996, GFI = 0.997, NNFI = 0.995, RMSEA = 0.032 (0.024–0.040), SRMR = 0.036].

#### 2.1.2 Study 2

##### 2.1.2.1 Sample

The participants (*N* = 494) were in-service teachers working in primary and secondary education. 21.5% were men, and 78.5% were women. The mean age was 44.8 years (*SD* = 9.49), and the teaching experience ranged from 14 to 26 years.

##### 2.1.2.2 Measurements

Data collection was conducted using a web-based self-completion questionnaire, the TRi-STEM scale (Papagiannopoulou et al., 2023), which assesses teachers’ readiness to implement Science, Technology, Engineering, and Mathematics (STEM) education. The dimensions, along with their reliability measures, were as follows: Affective conditions (*α* = 0.97/*ω* = 0.97), cognitive conditions (*α* = 0.98/*ω* = 0.98), self-efficacy (*α* = 0.93/*ω* = 0.93), and STEM commitment (*α* = 0.89/*ω* = 0.88). The fit indices indicated satisfactory results [*χ*^2^_(249)_ = 981.287, *p < 0*.001, *TLI* = 0.942, *CFI* = 0.948, *RMSEA = 0*.078, 95% CI (0.073–0.083), *SRMR = 0*.062, *GFI = 0*.993, *NNFI = 0*.942].

## 3 Taxometric analysis

Taxometrics analysis is not a simple, straightforward algorithm but includes multiple mathematical procedures that compare two alternative structures, taxon and non-taxon. The procedure known as *coherent cut kinetic* (Meehl, 1995) utilizes both numerical calculations and graphical representations. These are interpreted according to hypotheses and involve comparisons, providing researchers with suitable indices of consistency for decision-making (McGrath and Walters, 2012; Ruscio et al., 2013).

### 3.1 Taxometric graphs

The construction of taxometric graphs, based on the application of base rates or cut-off diagnosis, begins as follows: First, one variable is designated and named the *input* variable, while the others are labeled *output* variables. Subsequently, all cases are ordered according to the input variable and then divided into segments or cuts, referred to as “windows.” Subsequently, the output variables are subjected to various statistical operations that can provide insights into the structure of the latent constructs being studied (Meehl and Yonce, 1994; Ruscio et al., 2013). The most commonly used procedures are MAMBAC, MAXEIG, MAXCOV, and L-Mode, which are briefly described below.

*MAMBAC* stands for Mean Above-Minus Below A Cut. In this procedure, after sorting and partitioning the input variable with a series of cut points, the subsequent statistical operations on the output variables involve calculating the mean difference for scores above and below each cut point. If the number of variables k is ≥2, then k(k – 1) analyses are conducted to examine all possible input–output pairings. The corresponding MAMBAC diagram illustrates these mean difference sequences on the y-axis, while the x-axis represents the sorted cases. The plot varies for prototypical categorical data and dimensional data (Meehl and Yonce, 1996). In the former, distinct peaks appear adjacent to the cutting scores, while in the latter, the resulting plot takes on a “bowl-shaped” form.

*MAXCOV* stands for Maximum Covariance and includes one input and two output indicator variables. After sorting and partitioning the output variables, their covariance is calculated. The MAXCOV graph depicts the covariance plotted along the y-axis as a function of the mean scores of the input indicator. In this diagram, the change in covariance between the two indicators is shown as a function of the levels of the input variable. If the analysis is carried out with indicators k > 3, they are examined in triplets, producing k(k— 1)(k — 2)/2 corresponding curves (Meehl and Yonce, 1996). The MAXCOV curves obtained from prototypical dimensional data are flat because the covariance between the indicators remains almost constant due to shared loadings on the latent dimension. These differ from MAXCOV curves obtained from prototypical categorical data, which show apparent peaks with the maximum value placed within the subsamples.

*MAXEIG*: This refers to the Maximum Eigenvalue approach, where the largest eigenvalue of the covariance matrix is used. Consequently, it can incorporate all available variables in a single step (Waller and Meehl, 1998; Ruscio et al., 2013). The graph interpretation is similar to MAXCOV; however, MAXEIG is generally preferred and is used in the current analyses. When the procedure begins with one input indicator and k-1 output variables, k MAXEIG curves are produced, distinguishing categorical from dimensional data.

*L-Mode*: The L-Mode, or Latent Mode method, utilizes factor analysis and requires at least three variables. For the first component, the factor scores are estimated using Bartlett’s weighted least squares method, along with their frequency distribution curve (Waller and Meehl, 1998; Walters et al., 2010). It is anticipated that this distribution will be bimodal for categorical data and unimodal for dimensional data.

The three procedures—MAMBAC, MAXEIG, and L-Mode (MAXCOV excluded)—are available in *R* and were utilized in the current research. The differentiation of dimensional form taxon latent structures, along with their corresponding graphs and interpretations, can be easily demonstrated through TA analysis of artificial data (Stamovlasis et al., 2018).

The essential feature of the above procedures and the actual output is the graphical representations, which exhibit different patterns for the two potential latent structures and indicate taxon or dimensional cases. Each of the TA procedures is conceptually distinct and employs different mathematical manipulations, while all of them utilize multiple quantitative indicators of the latent variables under study. Given a chosen procedure, the researcher determines how to assign variables to input and output configurations, as well as the location of cutting scores and segments along input variables. The procedures are facilitated by simulation studies and the development of the CCFI (see next section), which allows for optimal decisions. Further details about the use and interpretation of the above graphical procedure can be found in recent presentations on TA (e.g., Ruscio and Wang, 2022).

### 3.2 The comparison curve fit index (CCFI)

The first step in TA is the construction of taxometric plots, which, at a glance, can be informative about the nature of the data under examination. However, this qualitative evaluation, based on irregularities, discontinuities, or distinct peaks, requires additional quantitative measurements to draw robust conclusions. The main statistical technique in TA relies on comparing graphs derived from empirical data with corresponding graphs generated from artificial data. These parallel analyses use simulated datasets created from the input empirical data, designed to maintain key characteristics such as the number of observable variables, sample size, marginal distributions, and correlation or covariance matrices. Consequently, the conclusions depend on the comparison between real and simulated data. The latter consists of bootstrapped datasets that originate from the former, representing the idealized categorical and dimensional structures that serve as the basis for comparison, while a quantitative index aids in determining the superior structure (dimensional vs. taxon). The validity and robustness of this approach have been supported by studies employing Monte Carlo methods (Ruscio and Kaczetow, 2009). The graphical representations are employed to compare the two competing structures. The key measure used to assess whether empirical data aligns with the “ideal” categorical or dimensional comparison data is the *Comparison Curve Fit Index* (CCFI). The CCFI necessitates the calculation of the *Root Mean Square Residual* (RMSR), which is found using Equation 1:


(1) $$RMSR_{\mathrm{cat}}={\left[\sum {\left(y_{emp}-y_{\mathrm{cat}}\right)}^{2}/N\right]}^{1/2}$$

*N* is the number of data points in the graph; (y_emp_ – y_cat_) is the distance between points for the empirical data (y_emp_) and the corresponding points for the “ideal” categorical comparison data (y_cat_). A perfect fit arises when the value of RMSR_cat_ equals zero. Similarly, for dimensional data, the corresponding measure RMSR_dim_ is defined and calculated accordingly.

Ergo, CCFI is defined by the Equation 2:


(2) $$\mathrm{CCFI}=\frac{RMSR_{\text{dim}}}{\left(\mathrm{RMSRca}t_{\text{dim}}\right)}$$

The CCFI index ranges between zero and unity. If CCFI >0.5 supports the taxon hypothesis, whereas a CCFI <0.5 indicates a dimensional structure. When the CCFI is exactly 0.5, or more reliably within the range 0.4 < CCFI<0.6, the structure is considered ambiguous.

The application of TA, in addition to the theoretical conjectures mentioned previously in the present study, requires practical presuppositions regarding the data (Meehl, 1995; Ruscio et al., 2010): (a) the sample size N should be ≥300; (b) The number of variables must be at least two (k ≥ 2); (c) the number of categories in the ordinal variables should consist of at least four (C ≥ 4); (d) the between-group validity of each variable must be d ≥ 1.25; (e) correlations among variables within groups formed during some intermediate stages of the TA procedure should be smaller than *r* ≤ 0.30. Information regarding the fulfillment of these requirements is included alongside the progression of calculations in the output of a TA, which can be easily performed in R using the *RTaxometrics* package. In the Supplementary material, we present the syntax and coding applied in R to conduct TA.

## 4 Results

**Figure 1:**
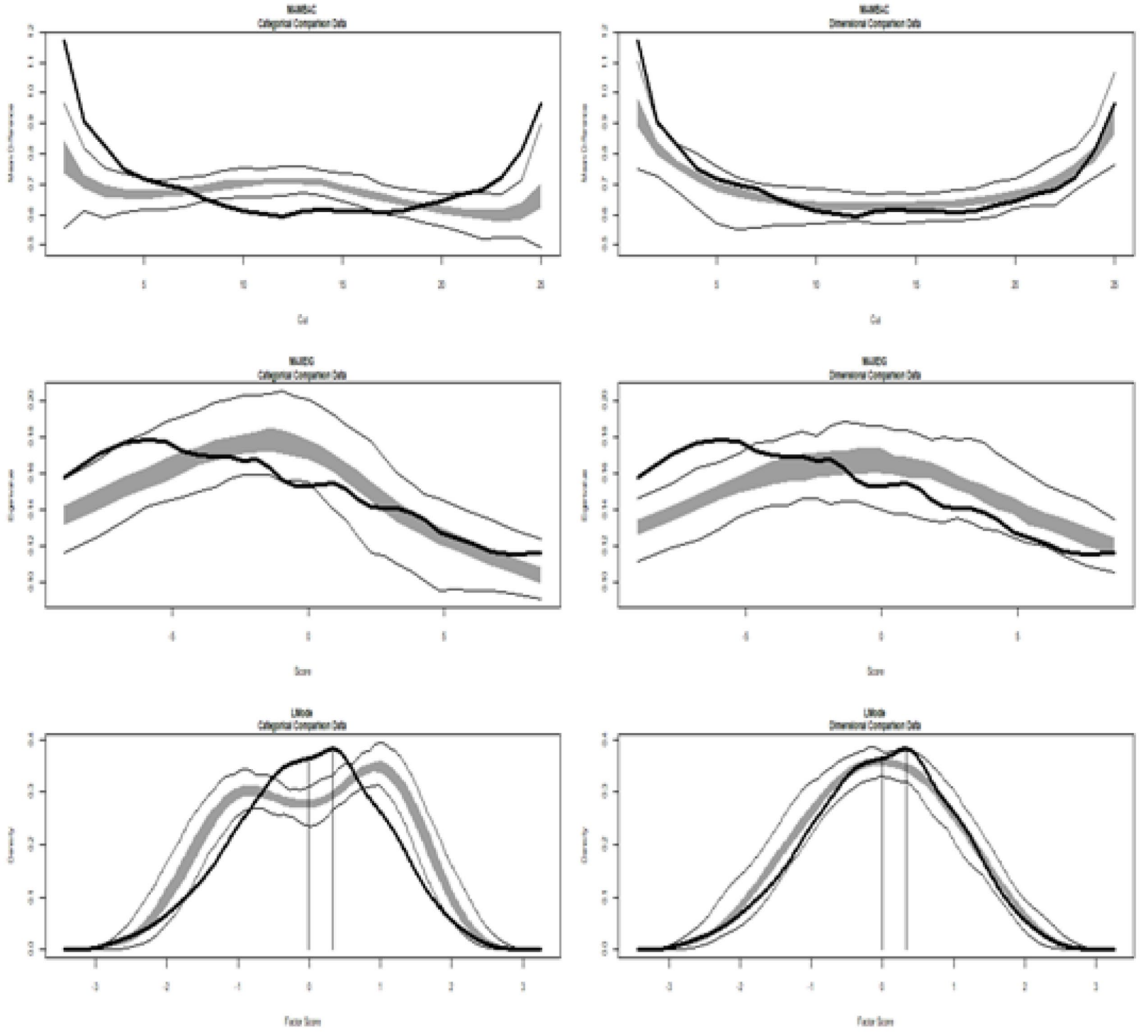
Input empirical data from Study 1: all achievement goals. (Left side) Comparison with categorical data. (Right side) Comparison with dimensional data. Results for MAMBAC (top), MAXEIG (middle), and L-Mode (bottom) analyses. Dark lines show the results for empirical data, and lighter lines show the results for parallel analyses of comparison data; the lines contain a band that spans ±1 SD from the mean at each data point on the curve.

**Figure 2:**
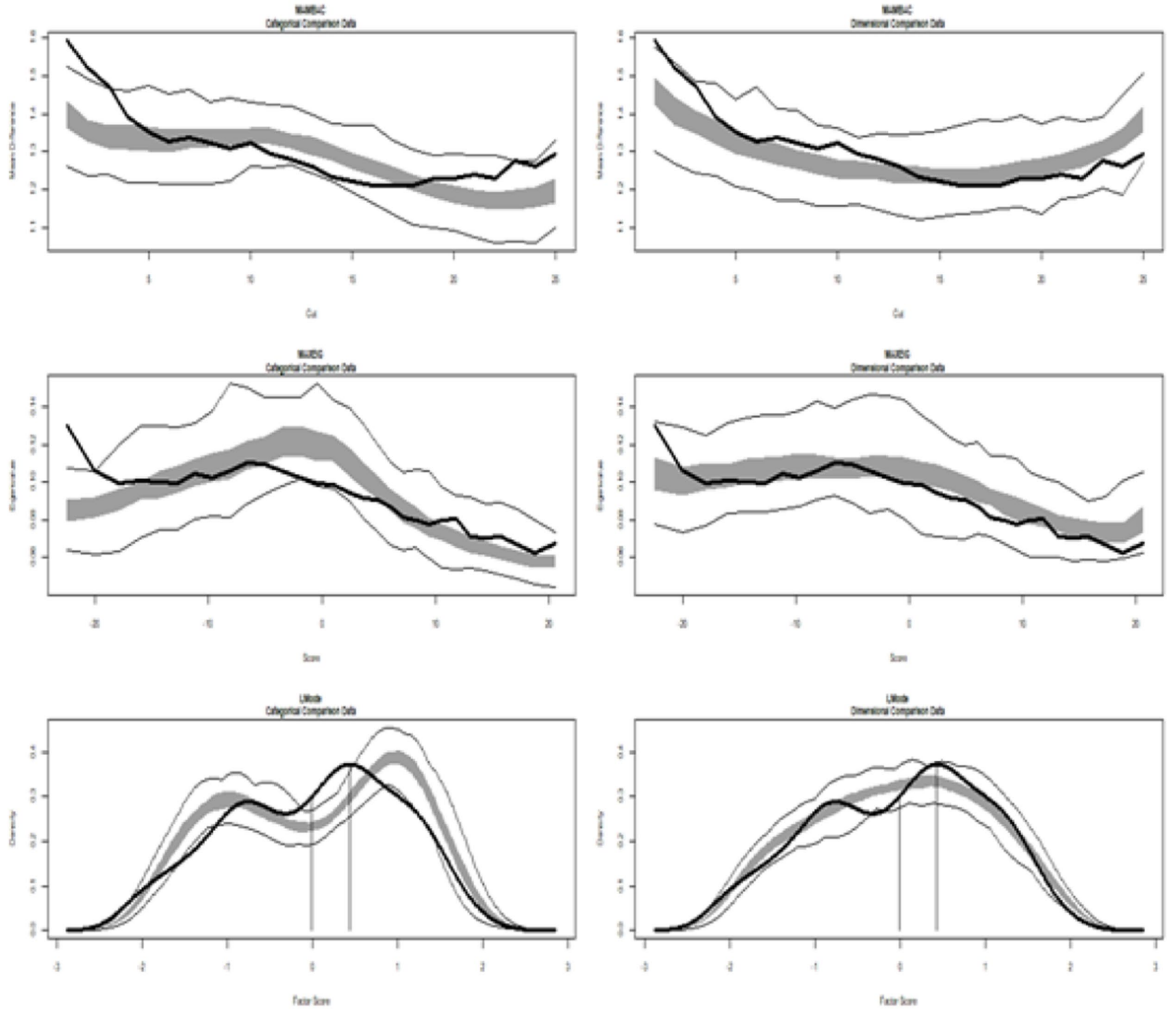
Input empirical data from Study 2: all dimensions of readiness. (Left side) Comparison with categorical data. (Right side) Comparison with dimensional data. Results for MAMBAC (top), MAXEIG (middle), and L-Mode (bottom) analyses. Dark lines show the results for empirical data, and lighter lines show the results for parallel analyses of comparison data; the lines contain a band that spans ±1 SD from the mean at each data point on the curve.

Table 1 (Study 1) and Table 2 (Study 2) show the results of TA, showing the values of the calculated CCFIs from which the conclusions are drawn. Moreover, the corresponding graphs, displayed in Figure 1 (Study 1) and Figure 2 (Study 2), illustrate the multidimensional constructs of Achievement Goals and Readiness, respectively (the corresponding taxometric graphs for each dimension are not provided).

**Table 1:** Comparison curve fit index (CCFI) for empirical data of Study 1.

		MAMBAC	MAXEIG	L-Mode	Mean
Empirical data of Study 1	All achievement goals	0.3007595	0.542394	0.2745595	0.372571
	Mastery approach	0.2765420	0.1800046	0.2632755	0.2399407
	Performance approach	0.2812768	0.2372808	0.2856836	0.2680804
	Performance avoidance	0.4020789	0.1945877	0.2455785	0.2807484
	Self-efficacy	0.1646637	0.1935441	0.182803	0.1803369

**Table 2:** Comparison curve fit index (CCFI) for empirical data of Study 2.

		MAMBAC	MAXEIG	L-Mode	Mean
Empirical data of Study 2	All dimensions of readiness	0.4298142	0.3868891	0.5339848	0.4502294
	Cognitive conditions	0.4404665	0.4832644	0.4743261	0.4660190
	Affective conditions	0.4479457	0.3629354	0.3884939	0.3997916
	Commitment	0.4641247	0.2985264	0.5209352	0.4278621
	Self-efficacy	0.2486682	0.3408031	0.379464	0.3229784
	Attitudes	0.2211668	0.5278131	0.3840924	0.3776908

### 4.1 Study 1

Figure 1 displays the graphs of MAMBAC, MAXEIG, and L-Mode analyses of the real data, including all achievement goals (mastery, performance approach, and performance avoidance), contrasted with idealized dimensional data (on the right) and categorical data (on the left). The MAMBAC graph is at the top, followed by MAXEIG in the middle and L-Mode at the bottom. A visual inspection of the compared curves does not support either a taxon or dimensional structure (Figure 1). There is no consensus among the three indices, and the mean value is 0.372, indicating that CCFI values support an ambiguous structure. The corresponding taxometric graph is shown in Figure 1.

Next, are the values of CCFIs for each dimension: Mastery (CCFIs: MAMBAC = 0.276, MAXEIG = 0.180, and L-Mode = 0.263, with a mean value of 0.239), Performance-approach (CCFIs: MAMBAC = 0.281, MAXEIG = 0.237, and L-Mode = 0.285, with a mean value of 0.268), Performance-avoidance (CCFIs: MAMBAC = 0.402, MAXEIG = 0.194, and L-Mode = 0.246, with a mean value of 0.281), and Self-efficacy (CCFIs: MAMBAC = 0.164, MAXEIG = 0.193, and L-Mode = 0.183, with a mean value of 0.180) (Table 1). In the cases of a single dimension, the latent construct appears to possess a dimensional structure except for performance avoidance in MAMBAC, which falls within the range of ambiguity (0.402).

The TA analysis in Study 1 suggests that some simple dimensions appear dimensional, but when we test the multidimensional achievement goals (including mastery, performance approach, and performance avoidance), the estimated CCF values fall into a zone of ambiguity.

### 4.2 Study 2

Figure 2 depicts the graphs of data from Study 2. The MAMBAC graph is positioned at the top, MAXEIG is in the center, and L-Mode is at the bottom. The analysis included all dimensions of teacher readiness for implementing STEM in education (Cognitive, Affective, Self-Efficacy, and Commitment), with their taxometric graphs displayed alongside idealized dimensional data (on the right) and categorical data (on the left). A visual inspection reveals differences in the graphical representations and highlights the values of the calculated CCFIs: MAMBAC = 0.430, MAXEIG = 0.387, and L-Mode = 0.534, with a mean of 0.450, which do not support a dimensional or taxon structure.

Table 2 shows the CCFIs for each dimension of readiness: Cognitive conditions (CCFIs: MAMBAC = 0.440, MAXEIG = 0.483, and L-Mode = 0.474 with a mean value of 0.466) clearly appear as an ambiguous structure. Affective conditions (CCFIs: MAMBAC = 0.448, MAXEIG = 0.363, and L-Mode = 0.388 with a mean value of 0.399) are very close to the zone of ambiguity, and the structure is considered ambiguous. Commitment (CCFIs: MAMBAC = 0.464, MAXEIG = 0.298, and L-Mode = 0.521 with a mean value of 0.427) constitutes a clearly ambiguous structure. Self-efficacy (CCFIs: MAMBAC = 0.249, MAXEIG = 0.341, and L-Mode = 0.379 with a mean value of 0.323) is a dimensional construct. Finally, in Attitudes (CCFIs: MAMBAC = 0.221, MAXEIG = 0.528, and L-Mode = 0.384 with a mean value of 0.378), the indices are not in agreement, and the mean value approaches the zone of ambiguity; thus, attitudes do not exhibit a clear structure.

The conclusion is analogous to the previous study: Some dimensions are clearly dimensional, and some have ambiguous structures, particularly when multidimensional constructs are examined.

## 5 Discussion

### 5.1 Interpretation of TA

Some additional comments should be made regarding the present analytic procedure, which concerns the taxometric inferential framework. The statistical method differs in that it does not aim to reject a null hypothesis (H_0_) but rather relies on the consistency among various mathematical procedures (Ruscio, 2007; Ruscio et al., 2011). The best interpretation involves considering the two competing hypotheses related to categorical and dimensional structures assessing support or rejection of one against the other. This evaluation is conducted through a comparison of the distinct features of taxonic and continuous data, *ceteris paribus* (sample size, number of indicators, correlations, etc.). To achieve this, Monte Carlo techniques and artificially simulated comparison data, which possess the same distinct features, provide valid support for effective decision-making (Ruscio and Kaczetow, 2009).

### 5.2 Limitations

The TA applied additional analyses from the available data and previously published studies. There are several limitations, of course, stemming from its exploratory nature, the opportunity sampling procedures, and possibly the usual sources of error involved in the measurement process. Other limitations arise from the unfulfilled requirements for TA implementation, which are related to a few violations and borderline values present in the data analysis. Nevertheless, simulation studies have shown that these violations could be counterbalanced by the other fulfilled criteria in the same data set (Ruscio et al., 2011). Additionally, CCFI reduces the chance of and prevents confirmation bias when interpreting the TA results (Ruscio and Kaczetow, 2009; Simmons et al., 2011). Furthermore, the nature of the latent constructs that were investigated may vary with sample type regarding individual characteristics, age, gender, and even cultural differences (Fiske, 2002), which were not examined here.

### 5.3 Methodological and epistemological implications

The nature of psychoeducational constructs remains an enduring puzzle for psychometrics. Provisionally, the problem is addressed by examining all alternative models (e.g., factor and latent class models) and making decisions based on fit-index comparisons. Auxiliary presuppositions about a hypothesized structure are helpful if they align with theoretical conjectures. This seemingly reasonable approach is, in fact, a heuristic solution. An issue arose and became a challenge when it was shown that many continuous-variable models have statistically equivalent categorical or mixture alternatives (Halpin et al., 2011). For instance, fitting a latent class model to empirical data does not ensure that the latent construct under study is categorical, as a continuous factor model can also fit the data (Molenaar and von Eye, 1994; Erosheva, 2005). This controversy may be justified by referencing classification errors; however, it might also be related to the ontological status of the latent variables themselves (Borsboom et al., 2003; Borsboom and Cramer, 2013; De Schryver et al., 2015; Epskamp et al., 2017). TA investigates this ontology.

TA aims to provide a definitive conclusion on the central hypothesis and can act complementarily to reinforce the structure in question. In practice, combining psychometric measurement models with taxometrics is an essential strategy (McGrath and Walters, 2012). Given that both traditional psychometric models and taxometrics, despite their limitations, are fundamentally robust methodological tools for research, their findings should be examined together, and the implications for theory and practice should be discussed.

Considering the above and the findings of the present research, we posited a problematization and suggested rethinking the nature of psychoeducational constructs. Analogous problematization, in light of taxometric results, as mentioned earlier, has already appeared in psychopathology research, where the identification of the nature of latent causes associated with various symptoms is an urgent need for defining the latent construct. In psychoeducational research, even though it might appear less imperative, the issue is crucial for theory development and for understanding changes in educational practices.

Taxometrics in this study revealed that some cases exhibited specific dimensional structures, while others were ambiguous. These ambiguous cases, characterized by an apparent ‘dual’ nature of these psychoeducational constructs, require further examination, suggesting that the latent construct may behave as continuous in some contexts and taxonic in others, depending on how we configure the measurement process. This duality is similar to the wave-particle duality in quantum mechanics, where matter appears as either a particle or a wave depending on the theoretical framework guiding the experimental setting.

Nonetheless, this metaphor is inappropriate for understanding psychoeducational constructs. A more realistic psychological interpretation is to consider that the nature of the latent construct’s structure varies among individuals. This contradicts the traditional assumption that all latent constructs are uniform, either categorical or continuous. Therefore, it is reasonable to accept that a latent construct functions as a categorical entity for some individuals while it could function as dimensional for others.

This interpretation in psychiatric research has led to questioning the traditional representation of latent constructs through established psychometric models that involve a presumed linear causal relationship between the hypothesized latent variable and the corresponding observable variables. This mathematical modeling and conceptualization of latent constructs fail to account for empirical findings in traditional analyses; moreover, non-linear changes are often observed. To this end, scholars have proposed a shift in theoretical thinking (Cramer et al., 2010, 2012a, 2012b), advocating for network representations of latent constructs. The theoretical framework promoted by network psychometrics describes an ontology of complex systems that better explain the relevant phenomena.

Moreover, in psychoeducation research, acknowledging that a latent construct may function as a categorical entity for some individuals while playing a dimensional role for others undermines the traditional ontological view of latent constructs as sole hypothetical entities that act as common causes of their manifestations. This conventional linear representation of latent variable theory aligns with *substance philosophy* (Seibt, 2022) and fails to explain TA findings and potential evidence of non-linear phenomena if such evidence exists. In other words, changes in the psychoeducation constructs of taxon structure can occur in a non-linear fashion and are observed as sudden, discontinuous shifts between distinct stages. It is crucial to note that the application of catastrophe theory models to the same data sets revealed evidence of non-linear phenomena; specifically, beyond a threshold value of the bifurcation variable, changes manifest as transitions (Stavropoulou et al., 2024; Vaiopoulou et al., 2024). This finding aligns with the present TA findings and the proposed interpretation.

Thus, we connect our problematization to the advancement of network psychometrics applied in psychiatric research (De Schryver et al., 2015; Epskamp et al., 2017). Notably, the network ontology provides a theoretical explanation by accommodating non-linear phenomena (van der Maas et al., 2020).

The ambiguous structures of latent constructs in TA were identified when examining multidimensional constructs, such as achievement goals or teachers’ readiness. This suggests that the interaction among dimensions leads to non-linear effects and the *emergence* of both types of latent structure among individuals at varying levels of complexity. Therefore, latent variable theory must align with *process philosophy* (Seibt, 2022) and the non-linear framework (van Geert and de Ruiter, 2022), where the structure of psychological constructs arises through the interaction between agents involved in the psychological process under study. The findings and interpretations in this endeavor regarding the implications for theory and measurement align with ongoing discussions about the limitations of traditional statistical measurement methods to address non-linear dynamical and multi-level relationships for consistent psychological traits (Trofimova et al., 2018; Sulis, 2022).

In conclusion, TA is a valuable tool for both traditional and modern psychometrics. This utility stems from its design and inherent aim to provide insights into the nature of a latent construct. Ordinary measurement models, such as factor models, LCA, or IRT, operate under prior assumptions regarding the nature of the variable in question, while the evaluation of the model relies on its calculated parameters and fit indices regarding the hypothesized structure. In contrast, TA provides a direct answer regarding the structure of the psychoeducational constructs in question, highlighting the paramount importance of its complementary application. The use of TA in this study, as mentioned in the rationale section and elaborated upon in the preceding discussion, extends beyond basic hypothesis testing. The present TA findings can be theoretically interpreted and associated with other major inquiries and methodological advantages, such as the anticipated modes of change in cognitive, attitudinal, and developmental variables, as well as potential non-linear phenomena. TA sparks epistemological reflections and discussions that are beneficial for the advancement of contemporary psychometric principles.

## Data Availability

The raw data supporting the conclusions of this article will be made available by the authors, without undue reservation.
