# Supplementary material for: On the structure of psychoeducational constructs: taxometric analysis and epistemological implications
Source: Front Psychol. 2025 Mar 17;16:1499960. doi: 10.3389/fpsyg.2025.1499960 (PMC11955598; doi:10.3389/fpsyg.2025.1499960)
Supplement: Supplementary file 1 [file Data_Sheet_1.PDF]

Supplementary Material

Using RTaxometrics package in R: Coding and Syntax

The Empirical Data are in the file: *Empirical-Data.txt*

placed in the folder named: “Taxon” on the Desktop

```
# Load the RTaxometrics package
#(The input data is in Empirical-Data.txt file)

> Data<-read.table("C:\\Users\\User\\Desktop\\“Taxon” \\Empirical-Data.txt", header=T)

> attach(Data)
> names (Data)
[1] "Q1" "Q2" "Q3" "Q4" "Q5" "Q6" "Q7" "Q8"     # reads the input variables (8 Variables)

>Data                                          # reads and types the input data

> x<-ClassifyCases(x, p=0.5, cols=1-8)         # Function preparing the data for
                                               taxometric analysis. It assigns cases to
                                                groups using the base-rate classification
                                               technique (x= input data matrix, p=base-
                                               rates used for classification,
                                                cols=columns containing data)

> CheckData(x)                                 # function checking the suitability of the input
                                                  empirical data for taxometric analysis,
                                               # the output provides the relevant information
                                                (distributional  characteristics, Cohen's d,
                                                  within-group correlations etc,)

>test.dim<-CreateData("dim")                   # creates prototypical dimensional data
> test.cat<-CreateData("cat")                  # creates prototypical categorical data

# RunTaxometric analysis that includes all functions.

> RunTaxometrics(x, seed=1,n.pop=100000, n.samples=100, reps=10, MAMBAC=TRUE,
assign.MAMBAC= 2, n.cuts=25, n.end=25, MAXEIG=TRUE, assign.MAXEIG=3,windows=30,
LMode=TRUE, mode.l=-0.001, mode.r=0.001,MAXSLOPE=TRUE)
```

(See also for further details in Ruscio (2017) or in Stamovlasis et. al, 2018, supplementary material with RTaxometrics-short tutorial)
